# Wartime stress and relapse risk in people with multiple sclerosis: a prospective cohort study

**DOI:** 10.1007/s00415-025-13366-9

**Published:** 2025-09-08

**Authors:** Roy Aloni, Carmit Dror, Tamar Barazani, Alon Kalron

**Affiliations:** 1 Department of Psychology, Ariel University, Ariel, Israel; 2 Multiple Sclerosis Center, Sheba Medical Center, Derech Sheba 2, Tel Hashomer, Israel; 3 Department of Physical Therapy, Gray Faculty of Medical and Health Sciences, Stanley Steyer School of Health Professions, Tel-Aviv University, Tel-Aviv, Israel; 4 Sagol School of Neuroscience, Tel-Aviv University, Tel-Aviv, Israel

**Keywords:** Multiple Sclerosis, War, Stress, Coping, Perceived fatigue

## Abstract

**Introduction:**

Psychological stress has been proposed as a trigger for disease activity in multiple sclerosis (MS), but findings have been inconsistent. While prior research has focused largely on chronic stressors, little is known about how people with MS (pwMS) cope with acute, large-scale stress events such as war.

**Objective:**

Examine the effects of wartime stress following the October 7, 2023 attack on disease activity in pwMS, and to assess whether emotional factors are associated with relapse risk during this period.

**Methods:**

Clinical data on relapses and disability progression were collected retrospectively for the year preceding October 7, 2023, and prospectively for the year following that date. Participants completed standardized questionnaires assessing stress, anxiety, depression, fatigue, and coping flexibility between April and June 2024.

**Results:**

From the 145 pwMS included in the prospective study, 38% experienced at least one relapse in the post-war year, compared to 23% in the year prior. Perceived fatigue was significantly higher among those who experienced relapses, while anxiety, depression, and perceived stress were not significantly associated with relapse frequency. Coping flexibility did not moderate the relationship between psychological distress and relapse count. No significant change was observed in disability progression across the two time periods.

**Conclusions:**

Wartime conditions were associated with increased relapse activity in pwMS. Fatigue may serve as a sensitive marker of disease vulnerability during stress. Coping flexibility, as measured in this study, did not appear to buffer the effects of psychological distress on relapse risk.

## Introduction

Worldwide, over 2.8 million individuals were living with multiple sclerosis (MS) in 2020, reflecting a global prevalence of approximately 35.9 per 100,000. In Israel, about 7,000 cases were reported at that time, corresponding to ~ 77.8 per 100,000, which is more than double the global average and consistent with prevalence rates observed in other developed countries [1–3]. The disease course is highly variable and shaped by environmental, genetic, and epigenetic factors [4–6]. Relapses and disease progression in people with multiple sclerosis (pwMS) remain largely unpredictable. Psychological stress, defined in the transactional model as the perception that demands exceed coping resources [7], has long been proposed as a trigger for exacerbations, yet early evidence was inconsistent; some studies reported a positive association between stress and relapse risk [8], while others did not replicate this finding [9]. More recently, high-quality syntheses [10, 11] offer stronger support for a link between stress and MS disease activity.

The recent review by von Drathen et al. [11] identified three studies examining the effects of war-related stress involving missile attacks [12–14]. Two studies [13, 14] from the 2006 Israel–Lebanon war reported a threefold increase in relapse rates associated with missile exposure. These studies relied on overlapping datasets, suggesting a robust but geographically specific link. In contrast, a smaller Israeli study during the 1991 Gulf War [12] found a reduction in relapse risk. However, it had a limited sample (*n* = 32), vague methodology, and lacked confounder control. Common limitations include inadequate adjustment for psychological comorbidities and inconsistent relapse measures, highlighting the need for more rigorous research on large-scale stressors like war.

On October 7, 2023, Hamas launched a coordinated assault on Israeli civilians and military posts, resulting in over 1,194 deaths (843 civilians), 4,834 injuries, and 243 hostages [15]. The attack triggered widespread psychological trauma, particularly among those directly affected. In January 2025, a study from Hadassah University Medical Center reported that among 93 pwMS monitored annually by MRI, the rate of subclinical disease activity more than doubled, from 11.8% to 24.7%, following the October 7, 2023, attack. Patients were four times more likely to develop new MRI lesions compared to previous years [16].

Although coping strategies in pwMS have been widely studied [17, 18], few have explored their relationship to relapse or disease progression during acute stress events such as war. Most prior research has focused on responses to chronic or daily stressors [19, 20], leaving a gap in understanding how patients cope during large-scale trauma. Only one study, following the 2006 Lebanon War [21], examined coping styles under acute wartime stress and found that a proactive, problem-focused approach was associated with reduced relapse risk. However, that study did not address longer-term outcomes or the ability to adapt coping styles across contexts. Coping flexibility, the capacity to shift between strategies such as goal-directed and emotion-focused coping based on situational demands, offers a more dynamic framework, yet remains unexamined in the context of wartime stress and disease activity in MS.

This study addressed several key objectives. First, it examined whether the wartime situation following the events of October 7, 2023, was associated with an increased risk of relapse or disease progression in pwMS, defined by an increase in the Expanded Disability Status Scale (EDSS) score. Second, it investigated whether emotional parameters (e.g., depression, anxiety, perceived fatigue, and stress) were related to relapse risk or enhanced disease progression. Finally, it examined whether coping flexibility moderated this relationship. As hypothesized, higher levels of subjective stress were expected to be associated with increased relapse risk and accelerated disease progression, while the use of adaptive coping strategies, such as problem-focused or emotion-regulation approaches, was anticipated to be linked with more favorable clinical outcomes.

## Materials and methods.

### Study design and participants

This study employed a hybrid retrospective–prospective observational cohort design. Participants were adults with a confirmed diagnosis of MS [22], all of whom were regularly followed at the Sheba Multiple Sclerosis Center, Tel Hashomer, Israel. Patients with progressive forms of MS were excluded, as the study aimed to focus on relapse activity and disease progression unrelated to the progressive course of the disease. Thus, the analysis was limited to individuals with the relapsing–remitting type of MS. The study was approved by the Sheba Medical Center Institutional Review Board (Ref. SMC-0727–23) and the Tel Aviv University Ethics Committee (Ref. 0008071–1). All participants provided written informed consent prior to enrollment.

### Study protocol and disease activity measures

Between April and June 2024, approximately six to eight months after October 7, 2023, eligible patients were invited to complete a single in-person self-report session. During this visit, participants completed validated questionnaires assessing subjective stress, psychological distress, and coping strategies. Written informed consent was obtained from all participants prior to enrollment. All questionnaires were completed in a single session at the Sheba Multiple Sclerosis Center and administered by a trained research assistant who is a registered psychologist.

**Fig. 1:**
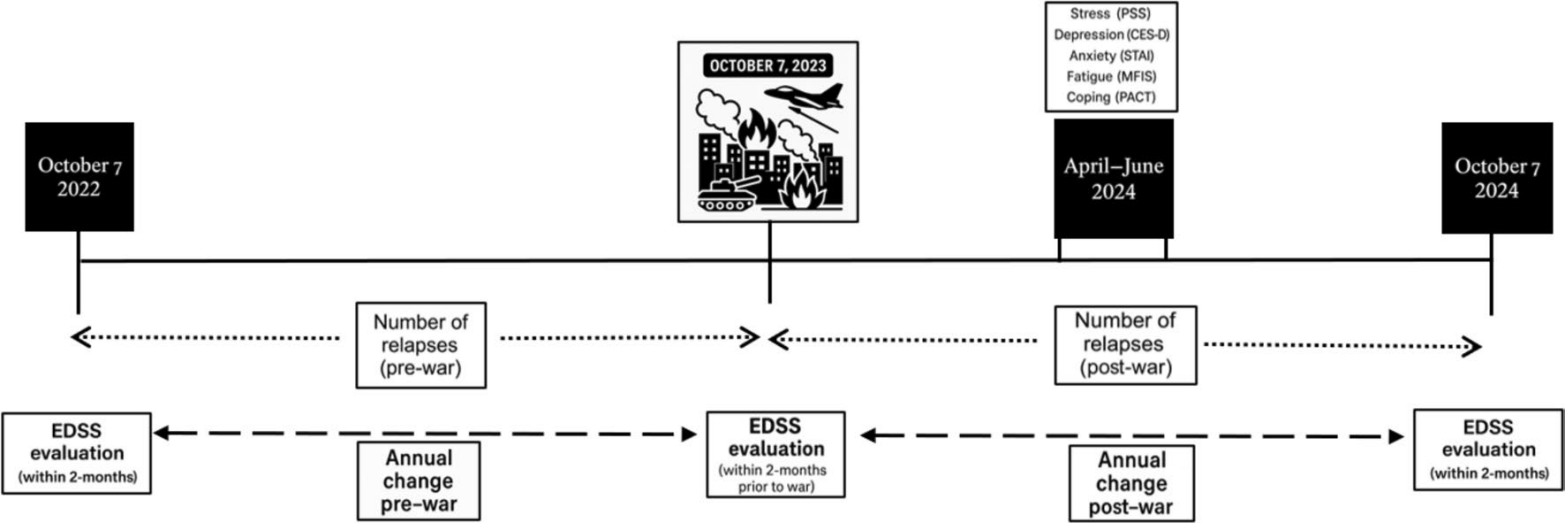
Study timeline and the corresponding assessment points

In parallel, clinical data were retrospectively extracted from electronic medical records to characterize each participant’s disease course in the year preceding the war. Specifically, we collected data on relapse frequency between October 7, 2022, and October 6, 2023, as well as EDSS scores recorded on October 7, 2022, and the closest available assessment prior to October 7, 2023 (within two months). Following questionnaire completion, participants were prospectively monitored through October 7, 2024. At the end of this follow-up period, we collected updated clinical data, including the number of relapses occurring between October 7, 2023, and October 7, 2024, and EDSS scores from the closest available visit before or after October 7, 2024 (within two months). Relapses were defined as new or worsening neurological symptoms lasting at least 24 h, in the absence of fever, infection, or other identifiable causes of pseudo-relapse, and were clinically verified and documented by a neurologist. Anamnestically reported episodes without clinical confirmation were not included. These timeframes were selected to enable a direct comparison of MS activity in the year before and after the October 7 war, in line with the study's primary objective. Figure. 1 presents the study timeline along with the assessment points.

### Stress, mood, and fatigue measures

Participants completed a series of standardized psychological measures to evaluate their emotional status. The State-Trait Anxiety Inventory (STAI) was administered to assess both state (situational) and trait (enduring) anxiety symptoms; this instrument has demonstrated strong validity in pwMS [23]. Depressive symptoms were measured using the Center for Epidemiologic Studies Depression Scale (CES-D), a 20-item tool validated in pwMS [24]. To capture participants' general appraisal of stress in daily life, defined as the perception that demands exceed coping resources [7], the Perceived Stress Scale (PSS) was utilized [25]. Given the high prevalence and clinical relevance of fatigue in the MS population, the study also included the Modified Fatigue Impact Scale (MFIS) [26], a self-report measure evaluating the perceived impact of fatigue across physical, cognitive, and psychosocial domains.

### Coping-related measure

Coping strategies were evaluated using the Perceived Ability to Cope with Trauma (PACT) scale [27], a validated self-report instrument that assesses individuals' perceived capacity to manage traumatic experiences. The PACT has been widely used in research involving highly trauma-exposed populations [28, 29]. It includes a measure of coping flexibility, which reflects the ability to engage with and shift between forward-focused coping (e.g., maintaining routines, pursuing goals) and trauma-focused coping (e.g., processing emotions, making meaning) based on situational demands. Higher scores indicate greater coping adaptability, which has been associated with improved psychological and physiological outcomes following trauma. For the present study, we used only the PACT Coping Flexibility score, as it provides a focused and theoretically grounded index of adaptive capacity relevant to understanding the occurrence of disease activity following the October 7th, 2023 war.

### Statistical analysis

Descriptive statistics were used to summarize the study sample’s demographic and clinical characteristics. The choice between parametric and non-parametric tests was based on the assessment of normality using the Shapiro–Wilk test. Missing data were handled using multiple imputation, which was conducted under the assumption that data were missing at random and allowed for the inclusion of all available cases in the analysis.

To examine whether disease activity differed between the year preceding October 7, 2023 and the year following it, we conducted a paired-samples t-test. The first comparison assessed the number of relapses in the year prior to October 7, 2023 versus the number of relapses in the year that followed. The second comparison evaluated changes in EDSS scores over the same periods: the difference in EDSS between October 7, 2022, and October 6, 2023 (pre-war) was compared to the difference between October 7, 2023, and October 7, 2024 (war).

A one-way ANOVA was conducted to examine the association between relapse frequency during the year following October 7, 2023, and levels of perceived stress, depression, anxiety, and perceived fatigue. The annual relapse rate was categorized into four groups: 0 (no relapses), 1, 2, and 3 relapses. When a significant main effect was detected, post hoc comparisons were performed using the Bonferroni test to adjust for multiple comparisons.

To examine whether coping flexibility moderated the associations between the number of relapses post October 7, 2023, and perceived fatigue, depression, anxiety, and perceived stress, we conducted a series of hierarchical linear regressions. Separate models were run for each variable. All continuous predictors were mean-centered prior to analysis to reduce multicollinearity. Interaction terms between each psychological variable and coping flexibility were computed and included in the models. In each regression, the number of relapses was entered as the dependent variable, with the psychological variable and coping flexibility entered in the first step, followed by the interaction term in the second step. A significant interaction term indicated a moderation effect, suggesting that the relationship between the psychological variable and post-treatment relapses differed depending on levels of coping flexibility. All analyses were performed using SPSS software (version 29.0 for Windows; SPSS Inc., Chicago, Illinois, USA). All reported p-values were two-tailed, with statistical significance at *p* < 0.05.

## Results

**Fig. 2:**
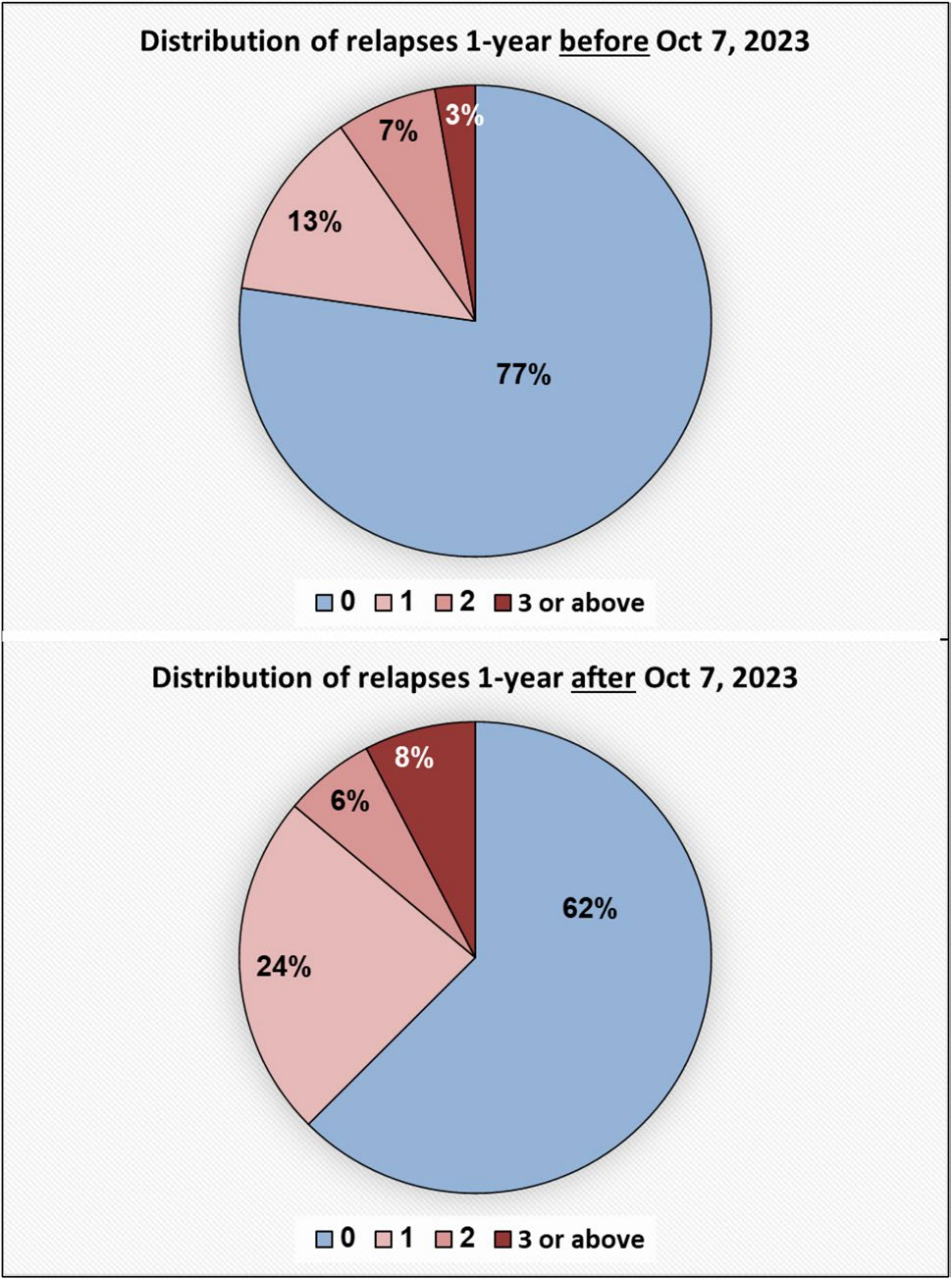
Distribution of annual relapses during the year before and after October 7, 2023 (n=145)

Data analysis included 145 pwMS (mean age = 39.9 years, SD = 13.8; 99 females; mean disease duration = 8.7 years, SD = 8.0). The mean number of relapses was significantly higher in the year following October 7, 2023, compared to the preceding year (0.59, SD = 0.91 vs. 0.37, SD = 0.80; *p* = 0.004). As shown in Fig. 2, 38% of participants experienced at least one relapse in the post-war year (2023–2024), compared to 23% in the year prior (2022–2023). However, no significant difference was observed in disability progression, as measured by annual change in EDSS scores: the mean increase was 0.51 in the year prior to October 7, 2023, and 0.55 in the following year (*p* = 0.241). Table 1 presents the demographic and clinical characteristics, usage of disease-modifying therapies and disease progression in the study cohort.

**Table 1:** Demographic and clinical characteristics, and disease progression in the study cohort (n = 145)

Variable	Mean (SD)		
Age (years)	39.9 (13.8)		
Sex (F/M)	99/46		
Disease duration (years)	8.7 (8.0)		
EDSS score on Oct 7, 2023	2.07 (1.8)		

Disease-modifying therapies (DMTs)		Pre-Oct 7, 2023 (n,%)	Post-Oct 7, 2023 (n,%)
Injectables			
Interferon beta-1a		2 (1.4%)	2 (1.4%)
Interferon beta-1b		1 (0.7%)	1 (0.7%)
Peginterferon beta-1a		2 (1.4%)	1 (0.7%)
Glatiramer acetate		3 (2.1%)	1 (0.7%)
Oral therapies			
Teriflunomide		5 (3.4%)	5 (3.4%)
Fumarates (Dimethyl fumarate, Diroximel fumarate)		13 (9.0%)	9 (6.2%)
S1P modulators (Fingolimod, Siponimod, Ozanimod, Ponesimod)		6 (4.1%)	7 (4.8%)
Cladribine		15 (10.3%)	21 (14.5%)
Infusions			
Natalizumab		36 (24.8%)	33 (22.8%)
Anti-CD20 therapies (Ocrelizumab, Ofatumumab, Ublituximab)		11 (7.6%)	18 (12.4%)
Alemtuzumab		1 (0.7%)	1 (0.7%)
No DMT		50 (34.5%)	46 (31.7%)

		p-value	Cohens' d
Annual change in EDSS (pre-Oct 7, 2023)	0.51 (0.50)	0.241	0.068
Annual change in EDSS (post-Oct 7, 2023)	0.55 (0.50)		
Annual number of relapses (pre-Oct 7, 2023)	0.37 (0.80)	0.004*	0.222
Annual number of relapses (post-Oct 7, 2023)	0.59 (0.91)		

*p*-value < 0.005

**Fig. 3:**
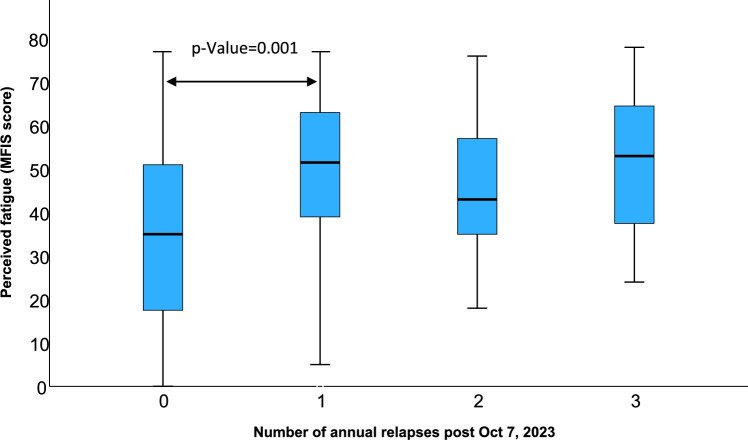
Perceived fatigue scores by relapse subgroup

During the one-year period following October 7, 2023, 34 had one relapse, 9 had two relapses, 11 had three relapses, and 91 did not experience a relapse. No differences between relapse subgroups were demonstrated in age (*p* = 0.949), sex distribution (*p* = 0.354), and EDSS (on Oct 7, 2023) (*p* = 0.211). Table 2 presents scores for perceived stress, depression, anxiety, and perceived fatigue across the relapse subgroups. Significant differences emerged in levels of perceived fatigue, depression, and perceived stress between the relapse subgroups. Bonferroni post hoc analysis revealed that a statistically significant difference was observed only in perceived fatigue, specifically between pwMS who experienced a single relapse and those who did not experience any relapse. PwMS who experienced a single relapse reported approximately 40% higher than those who remained relapse-free during the same period. Figure 3 illustrates perceived fatigue scores by relapse subgroup. No differences between relapse subgroups were demonstrated in anxiety and coping flexibility scores.

**Table 2:** Psychological and behavioral measures by number of relapses in the year following October 7, 2023

Variable	Number of annual relapses post Oct 7, 2023				*P*-value (Effect size)
	0 (*n* = 91)	1 (*n* = 34)	2 (*n* = 9)	3 (*n* = 11)	
Perceived fatigue	34.9 (20.7)^a^	49.0 (20.5)^b^	45.2 (18.3)^a,b^	51.3 (17.7)^b^	.001* (.104)
Depression	13.0 (6.9)^a^	15.7 (7.0)^a^	17.4 (7.2)^a^	17.4 (7.3)^a^	.047* (.055)
Anxiety	14.9 (4.2)	16.2 (3.9)	15.0 (3.3)	16.4 (2.6)	.314 (.025)
Perceived stress	31.2 (6.4)^a^	34.3 (6.7)^a^	30.8 (8.9)^a^	35.3 (5.6)^a^	.046* (.055)
Coping flexibility	4.5 (1.7)	4.7 (1.6)	4.1 (1.4)	5.4 (1.9)	.347 (.023)

^*^p-value < 0.05; superscript letters (a,b) denote significant pairwise differences based on Bonferroni post-hoc analysis

Hierarchical linear regression analyses were conducted to examine whether coping flexibility moderated the associations between psychological variables (perceived fatigue, depression, anxiety, and perceived stress) and the number of relapses following October 7, 2023. In all four models, the interaction terms between each psychological variable and coping flexibility were non-significant: fatigue (*p* = 0.444), depression (*p* = 0.244), anxiety (*p* = 0.438), and perceived stress (*p* = 0.583). These results indicate that coping flexibility did not moderate the relationships between psychological distress and relapse count, suggesting that the strength or direction of these associations did not vary as a function of coping flexibility.

## Discussion

This study aimed to investigate the impact of the wartime situation following October 7, 2023, on disease outcomes in pwMS, specifically examining relapse rates, disability progression, emotional parameters, and coping flexibility. Our key findings indicate a significant increase in relapse frequency during the year following the wartime event, with 38% of pwMS experiencing at least one relapse compared to 23% in the preceding year. However, no significant change was observed in disability progression as measured by EDSS scores.

Our results regarding increased relapse risk associated with war-related stress align with findings from previous studies conducted during the 2006 Lebanon war [13, 14] and following the first Gulf War [12]. Similar to our study, Golan et al. [13] and Yamut et al. [14] reported elevated MS relapse frequencies in periods associated with wartime stress. Notably, these studies included MS populations from both sides of the conflict (Israel and Lebanon), highlighting that the stress accompanying warfare itself is harmful regardless of being on the "winning" or "losing" side. Despite this similarity, a key difference between our study and prior research pertains to the timeframe of follow-up. Golan et al. [13] monitored participants during intervals of 33 days (equal to the duration of the 2006 Lebanon war), starting one year prior to the conflict and continuing up to three months afterward, reporting increased relapse rates specifically during wartime compared to non-war periods. Yamut et al. [14] adopted similar short monitoring intervals but extended their total observation period to three years (1.5 years before and after the war). Given the prolonged nature of the conflict following October 7, 2023, we utilized a continuous one-year follow-up period.

The variability in follow-up durations across these studies likely captures distinct aspects of stress-related disease activity. Shorter follow-up periods, such as those used by Golan et al. [13] and Yamut et al. [14], predominantly highlight acute stress responses and immediate relapse risk. In contrast, the extended timeframe employed in our study provides insights into both immediate and delayed or cumulative effects of prolonged wartime stress, reflecting chronic stress exposure. These methodological differences underscore the complexity involved in understanding the impact of wartime stress on MS disease activity and highlight the importance of selecting appropriate timeframes to adequately capture both short-term and sustained clinical effects.

Furthermore, unlike previous studies that focused exclusively on relapse frequency, our study also evaluated disease activity through monitoring changes in disability levels, measured by EDSS scores, over the full wartime year compared to the preceding year. Interestingly, we observed no significant differences in disability progression between these periods. One plausible explanation is that a one-year period may not be sufficiently long to detect meaningful changes in disability, as prior studies suggest that EDSS progression often requires several years of follow-up to become apparent [1, 5]. Additionally, it is possible that increased relapse frequency within a single year may not directly translate into measurable disability progression, especially when relapses are mild or effectively managed with disease-modifying therapies [6, 20]. Therefore, EDSS scores may lack the sensitivity needed to capture subtle or transient changes resulting from acute stress, reinforcing the need for longer-term monitoring to comprehensively assess the impact of prolonged wartime stress on MS disability outcomes.

A novel finding of this study was the association between relapses during wartime and perceived fatigue, while no significant relationship emerged with other psychological metrics, such as anxiety. The unique predictive value of fatigue may stem from its dual nature, encompassing both psychological distress and physiological exhaustion, potentially making it a more sensitive indicator of stress-induced disease activity in MS populations [30]. Similar to depression and anxiety, fatigue can also be directly related to underlying inflammatory or neuroimmunological processes, commonly implicated in MS pathophysiology, which may become exacerbated during prolonged periods of stress [31]. Consequently, fatigue might serve as an early marker of subclinical disease activity, not as ‘more physiological’ but as potentially capturing both transient fluctuations in physical energy and emotional strain, effectively bridging subjective emotional experience with objective physiological manifestations of stress. This highlights the clinical relevance of routinely assessing perceived fatigue in pwMS, particularly during periods of heightened stress exposure. In this study, we used only the total MFIS score, which was predefined as the primary fatigue measure and is widely applied as a global index of fatigue in pwMS. Subdomains of fatigue (cognitive, physical, psychosocial) were not analyzed, in part to avoid collinearity and type I error inflation given their high intercorrelations and our sample size. Future studies should incorporate subdomain-level and longitudinal fatigue assessments, as emphasized by Palotai et al. (2020) [32].

In contrast to the findings of Somer et al. [21], which indicated that a direct, problem-focused coping style was associated with reduced relapse risk among pwMS in a war zone, the present study did not find evidence that coping flexibility moderated the associations between fatigue, depression, anxiety, or perceived stress and the number of relapses following October 7, 2023. Several factors may account for this discrepancy. First, Somer et al. examined the role of specific, action-oriented coping strategies (e.g., direct planning, proactive behavior) during a defined period of acute wartime stress, whereas our measure of coping flexibility captured a broader, trait-like capacity to adapt coping styles across situations. It is possible that in the face of acute crises, concrete and targeted coping behaviors exert a more direct influence on relapse risk than general coping adaptability. Second, the contextual differences between the studies may be meaningful: while the prior study focused on a clearly demarcated war-related event, our study encompassed a more variable post-October 7 period with potentially diverse types and levels of stress exposure. Differences in the intensity and nature of the stressor, as well as the timing of coping assessment in relation to relapse occurrences, may have contributed to the divergent findings. Alternatively, the most parsimonious explanation is that coping flexibility simply does not relate to relapse activity. While coping strategies remain important for psychological adjustment, they should not be framed as determinants of biological disease activity, so as to avoid implying patient blame. Future research should further explore the role of specific coping strategies, particularly those oriented toward direct action, in influencing psychological well-being during periods of heightened stress.

The study is novel due to the relatively rare war situation; however, several limitations should be acknowledged. First, MRI markers of disease activity were not included, potentially omitting important neurobiological insights. Second, the war that began on October 7, 2023, has been prolonged and characterized by multiple intensity peaks, and our study could not differentiate between these varying dynamics. Third, we relied primarily on self-report measures (e.g., MFIS, CESD, STAI, PSS), which could introduce response biases. Additionally, the absence of baseline assessments prior to the war limits our ability to fully interpret the observed changes. Finally, given the mixed retrospective–prospective design, relapse data from the pre-war year were collected retrospectively, whereas post-war relapses were prospectively monitored, which may have introduced underreporting bias in the pre-war period. Because the war was unanticipated, a fully prospective design was not feasible. Moreover, exact visit dates for EDSS assessments, relapse onset, and questionnaire completion were not extracted into the analytic dataset, limiting our ability to evaluate temporal clustering of relapses or the precise interval between EDSS and questionnaire assessments. Future research should systematically incorporate such data to provide a more detailed understanding of the temporal dynamics of disease activity. Future research should consider routinely collecting these psychological metrics during regular patient follow-up visits, enabling a more nuanced understanding of their dynamics during extraordinary situations.

## Conclusion

This study provides evidence that the wartime context following October 7, 2023, was associated with increased relapse frequency in people with relapsing–remitting MS, underscoring the clinical relevance of large-scale stress exposure. Perceived fatigue emerged as a significant psychological correlate of relapse risk. However, coping flexibility did not moderate the relationship between psychological distress and disease activity. Future research should investigate which coping mechanisms most effectively reduce relapse risk under acute or sustained stress, ideally incorporating neuroimaging and real-time stress assessments. It is also important to examine whether observed relationships are influenced by the type or effectiveness of immunomodulatory treatment, as pharmacological factors may interact with psychological stress in shaping disease outcomes.

## Conflicts of interests

None.

## Ethical approval

The study was approved by the Sheba Medical Center Institutional Review Board (Ref. SMC-0727–23) and the Tel Aviv University Ethics Committee (Ref. 0008071–1). All participants provided written informed consent prior to enrollment.

## Data Availability

Data are available upon reasonable request.
